# Acute Alcohol Use and Suicide

**DOI:** 10.1001/jamanetworkopen.2024.61409

**Published:** 2025-02-24

**Authors:** Minkyung Yim, Hayoung Kim, Gyumyoung Kim, Ji-Won Hur

**Affiliations:** 1 School of Psychology, Korea University, Seoul, Republic of Korea

## Abstract

**Importance:**

While acute alcohol use (AAU) is known to increase the risk of suicide significantly, the underlying mechanisms of this association are still understudied.

**Objective:**

To examine the association between AAU and the risk factors for suicide, focusing on the association between AAU and choice of suicide methods.

**Design, Setting, and Participants:**

This cross-sectional study included data from the Korean National Investigations of Suicide Victims Study (KNIGHTS). The KNIGHTS dataset comprises nationally representative data encompassing nearly all suicide deaths in South Korea from January 1, 2013, to December 31, 2020. The study was analyzed from November 2 to 10, 2023.

**Main Outcomes and Measures:**

The main outcome was whether decedents who died by suicide had consumed alcohol before their death. Trained investigators conducted psychological autopsies by reviewing police reports of suicide deaths. Whether the decedent who died by suicide had consumed alcohol before death was determined from 3 sources: (1) confirmation by informants, (2) autopsy reports indicating a positive blood alcohol concentration, and (3) observations from police officers. Demographic characteristics, premortem psychiatric symptoms, and suicide characteristics, including method of suicide and presumed reasons for suicide, were examined. Hierarchical logistic regression models were used to examine the association between the contributing factors and AAU.

**Results:**

This study included 55 226 decedents who died by suicide (65.5% male), of whom 21 998 (39.8%) were under the influence of alcohol at the time of death. AAU was associated with being male (odds ratio [OR], 1.37 [95% CI, 1.31-1.44]); being middle aged (eg, from age 30 to 39 years: OR, 1.20 [95% CI, 1.06-1.35]); and having alcohol use disorder symptoms (OR, 13.28 [95% CI, 12.38-14.24]). Among suicide methods, gas, drug, and pesticide poisoning showed a positive association with AAU, with gas poisoning showing the highest OR (OR, 1.88 [95% CI, 1.61-2.20]). An interaction was found between age and suicide method, with older adults (eg, aged 80 years or older) having higher odds of AAU when using methods such as drug (OR, 6.28 [95% CI, 3.53-11.17]), pesticide (OR, 6.56 [95% CI, 3.86-11.13]), and gas (OR, 2.48 [95% CI, 1.52-4.04]) poisoning.

**Conclusions and Relevance:**

The findings of this cross-sectional study suggest that AAU was associated with gas poisoning, as indicated by the ORs. Given that charcoal burning involves substantial preparation time, alcohol may have been deliberately used to facilitate planned suicide attempts. Furthermore, the deaths of older adults who consumed alcohol prior to suicide, even when using less lethal methods, highlight the critical need for monitoring and effectively managing alcohol use within this population.

## Key Points

**Question:**

How is acute alcohol use (AAU) before suicide associated with the choice of suicide method?

**Findings:**

In this cross-sectional study of 55 226 suicide deaths in South Korea from 2013 to 2020, AAU was associated with gas poisoning, followed by drug and pesticide poisoning. In older adults, AAU was associated with less lethal methods.

**Meaning:**

The finding that AAU was associated with gas poisoning, which requires lengthy preparation, suggests the possibility of intentional alcohol use to facilitate suicide and warrants further investigation.

## Teaser

This cross-sectional study examines the association between acute alcohol use and risk factors for suicide among decedents in South Korea, focusing on the association between acute alcohol use and choice of suicide methods.

## Introduction

Acute alcohol use (AAU) is a recognized risk factor for suicide, as it significantly increases the likelihood of suicide attempts and deaths,^1,2,3^ even after accounting for alcohol use disorders (AUDs).^4^ AAU is defined as alcohol consumption within a few hours prior to a suicide attempt (eg, within 3 to 6 hours) or as the presence of positive blood alcohol concentration (BAC) in individuals who die by suicide.^5^ Between 21.0% and 44.4% of decedents who die by suicide worldwide have positive BAC levels.^6,7,8,9^ A meta-analysis found that AAU increased the risk of suicide attempts 7-fold and showed a dose-response association between alcohol levels and suicide risk.^3^

Although it is known that AAU increases the risk of suicide, the mechanisms by which it increases suicide attempt lethality remain unclear. Existing theoretical models on alcohol use and suicide behavior primarily conceptualize AAU as a proximal risk factor for suicide.^10,11^ By exacerbating psychologic distress and impulsivity while lowering inhibitions,^11^ AAU increases the likelihood of harmful decisions in moments of crisis.^12,13^ This may partially explain its associations with more lethal suicide methods. Some studies have reported associations between AAU and more lethal suicide methods, such as hanging or suffocation.^6,8,14,15^ In countries with legal gun ownership, AAU is associated with firearm suicides,^16^ the method with the highest case fatality rate.^17^ Positive BAC is shown to be more prevalent in individuals using high lethality methods, including self-immolation, burns, or electrocution, ranging from 43% to 67%, compared with lower lethality methods, such as stabbing, cutting, or piercing (7% to 16%).^18^

Conversely, some studies have reported no associations or nonlinear associations between AAU and suicide methods. For instance, 1 study reported that BAC was not associated with the choice of high lethality suicide methods, suggesting that AAU may not always lead to lethal method selection.^19^ Similarly, an autopsy study^20^ showed a nonlinear, bell-shaped association, indicating that the association of AAU with suicide method lethality is complex and not straightforward. Moreover, another study suggested an association of AAU with less lethal methods.^21^ These findings underscore the challenging complexity of the association between AAU and suicide method selection and the need for further research to understand this complexity through in-depth studies of demographic characteristics, psychiatric symptoms, and suicide characteristics.

The interaction among age, AAU, and suicide methods adds another layer of complexity. Older decedents who die by suicide typically have lower rates of positive BAC than their younger and middle-aged counterparts^6,8,22^ and tend to favor less lethal methods, such as drug poisoning.^23^ For example, older individuals who consumed alcohol before suicide are more likely to use drug poisoning and less likely to use a firearm.^24^ This suggests that age plays a significant role in the association between AAU and suicide methods.

It is also important to investigate the association between AUD and AAU to distinguish their respective association with suicide risk.^25,26^ AUD and AAU, although related, exhibit distinct characteristics in relation to suicide attempts. As a chronic condition, AUD serves as a distal risk factor for suicide,^10^ often accompanied by other risk factors for suicide, including depressive disorders and adverse life events,^25,26^ further elevating suicide risk.^27,28,29^ In contrast, AAU is more closely linked to the immediate circumstances surrounding a suicide attempt. This proximal and time-limited condition may exacerbate impulsivity.^12,13^ Furthermore, individuals with AUD compared with those with AAU exhibit distinct patterns in their choice of suicide methods; individuals with AUD are more likely to select less lethal methods compared with those with AAU.^29,30^ Therefore, it is necessary to consider both AUD and AAU in a single model to elucidate their intertwined associations with suicide.

Building on these understandings, we comprehensively examined AAU and suicide methods using nationally representative data from South Korea. In South Korea, highly lethal methods, such as hanging, jumping, and pesticide poisoning, are commonly observed in suicide deaths.^31^ We hypothesized that the association between AAU and these suicide methods would be influenced by demographic characteristics, premortem psychiatric symptoms including AUD, and other suicide-related factors. Using hierarchical logistic regression analysis, we assessed the associations between these variables and AAU. The potential implications of our findings for suicide prevention strategies and public health policies regarding alcohol underscore the significance of this study.

## Methods

### Data Source

This cross-sectional study included data from the Korean National Investigations of Suicide Victims Study (KNIGHTS) from January 1, 2013, to December 31, 2020.^32^ Trained investigators, comprising mental health professionals such as psychiatrists, mental health nurses, psychologists, and social workers experienced in psychiatric epidemiologic surveys, examined police reports on suicides from 254 police stations across 17 regions in South Korea. The investigators conducted psychological autopsies using the Korean Psychological Autopsy Checklist for Police Records^33^ for a comprehensive understanding of each individual. Further details regarding the KNIGHTS data are available elsewhere.^32,34^ The Institutional Review Board of Korea University approved this study, and informed consent was waived because the KNIGHTS data were deidentified. This study followed the Strengthening the Reporting of Observational Studies in Epidemiology (STROBE) reporting guideline.

### Measures

The primary outcome of interest was whether the decedent had consumed alcohol before death by suicide; the data were accordingly categorized as either the AAU or the control group. Determination was based on 3 sources: (1) informant confirmation of AAU before the suicide attempt; (2) autopsy reports with a positive BAC; and (3) observations from police reports, such as photographs of the suicide scene showing signs of drinking (eg, open or empty bottles).^34^ AAU was coded as yes, no, or unknown, and individuals for whom AAU was coded as unknown were excluded from the analysis. We excluded individuals for whom it was unclear whether they had been drinking immediately before death; the methods used in the suicide death were unclear; there was insufficient information to determine the presence or absence of psychiatric symptoms, including AUD; or the presumed reason for the suicide attempt was unknown. The independent variables entered into the model had 3 categories: demographic characteristics, psychiatric symptoms, and suicide characteristics.

The demographic variables included sex, age, and marital status. Psychiatric symptoms (AUD, depression, manic symptoms, psychosis, acute stress, and other substance use) were identified based on a comprehensive evaluation of multiple sources. These sources included family reports of the decedent’s medical history, observed symptoms prior to death, and available medical records. Variables were coded as yes, no, or unknown. Suicide characteristics included the method of suicide and presumed reasons for suicide. Suicide methods comprised 8 categories: hanging, drug poisoning, pesticide poisoning, gas poisoning, drowning, jumping, self-harm, and other. Suicide methods coded as unknown or other were grouped into a single other category. The other category included heterogeneous methods lacking detailed coding (eg, rare poisons, self-immolation, or firearms), while the unknown category provided insufficient information to determine the method of suicide. In suicide deaths with multiple methods, the most lethal method was determined based on police and autopsy reports. The presumed reasons for suicide consisted of stress-related circumstances that may have influenced suicide attempts, such as job stress, financial stress, family stress, interpersonal stress, physical and mental health problems, and other and unknown reasons.

### Statistical Analysis

Data were analyzed from November 2 to 10, 2023. A hierarchical logistic regression analysis was performed to examine the factors associated with AAU. Independent variables were added in sequential steps to assess their incremental associations with AAU likelihood. Model 1 included demographic variables such as age, sex, and marital status; model 2 included psychiatric symptoms before death; and model 3 included suicide characteristics to examine the associations between suicide methods and the presumed reasons for suicide. Finally, we adjusted for the interaction between age and suicide method in the final model (model 4). The odds ratios (ORs) were calculated with 95% CIs. Additionally, χ^2^ tests with *df*s were conducted to examine the association between age and the use of each suicide method among participants in the AAU group. Statistical significance was set at 2-sided *P* < .01. Analyses were performed using SPSS Statistics, version 27.0 (IBM Inc).

## Results

### Characteristics of Decedents Who Died by Suicide and Suicide Behaviors

Among the 102 922 suicide deaths in the dataset, we excluded 30 498 individuals for whom it was unclear whether drinking had occurred immediately before death; 75 for whom the methods used in the suicide death were unclear; 15 960 for whom there was insufficient information to determine the presence or absence of psychiatric symptoms, including AUD; and 1163 for whom the presumed reason for the suicide attempt was unknown. We analyzed data from the remaining 55 226 decedents who died by suicide in South Korea (34.5% female and 65.5% male) (Table 1). Among these individuals, 21 998 (39.8%) had consumed alcohol before death. The AAU group had a higher proportion of males (73.0%), and middle-aged individuals in their 40s and 50s accounted for 49.4% of AAU suicides. Regarding psychiatric symptoms before death, 33.8% of the AAU group reported symptoms of AUD, compared with only 3.6% of the control group. Among individuals who had symptoms of AUD before death, 86.5% belonged to the AAU group.

**Table 1.** Characteristics of Decedents Who Died by Suicide With or Without AAU

Characteristic	Suicide death, No. (%)		χ^2^ (*df*)	*P* value
	With AAU (n = 21 998)	Without AAU (n = 33 228)		
Sex				
Female	5940 (27.0)	13 099 (39.4)	903.68 (1)	<.001
Male	16 058 (73.0)	20 129 (60.6)		
Age, y				
<20	235 (1.1)	996 (3.0)	4603.09 (7)	<.001
20-29	2704 (9.4)	2940 (8.8)		
30-39	4105 (18.7)	3962 (11.9)		
40-49	5409 (24.6)	5008 (15.1)		
50-59	5457 (24.8)	5571 (16.8)		
60-69	2711 (12.3)	4656 (14.0)		
70-79	1515 (6.9)	5707 (17.2)		
≥80	492 (2.2)	4388 (13.2)		
Marital status				
Married	8613 (39.2)	14 509 (43.7)	2016.37 (5)	<.001
Separated	1326 (6.0)	1013 (3.0)		
Not married	6021 (27.4)	8336 (25.1)		
Widowed	815 (3.7)	3379 (10.2)		
Divorced	4052 (18.4)	3236 (9.7)		
Unknown	1171 (5.3)	2755 (8.3)		
Psychiatric symptoms				
Alcohol use disorder	7441 (33.8)	1206 (3.6)	9138.69 (1)	<.001
Depression	14 054 (63.9)	22 630 (68.1)	105.57 (1)	<.001
Anxiety disorder	25868 (11.8)	4455 (13.4)	32.46 (1)	<.001
Manic symptoms	456 (2.1)	1027 (3.1)	52.47 (1)	<.001
Psychosis	700 (3.2)	3124 (9.4)	794.44 (1)	<.001
Acute stress	2117 (9.6)	2469 (7.4)	83.61 (1)	<.001
Other substance use	396 (1.8)	181 (0.5)	201.78 (1)	<.001
Suicide methods				
Hanging	10 790 (49.0)	16 765 (50.5)	6252.81 (7)	<.001
Drug^a^	988 (4.5)	912 (2.7)		
Pesticide^b^	2064 (9.4)	3140 (9.4)		
Gas^c^	5013 (22.8)	3436 (10.3)		
Drowning	388 (1.8)	659 (2.0)		
Jumping	2313 (10.5)	7625 (22.9)		
Self-harm^d^	222 (1.0)	490 (1.5)		
Other^e^	220 (1.0)	201 (0.6)		
Presumed reason for suicide				
Job stress	879 (4.0)	1296 (3.9)	4219.05 (6)	<.001
Financial stress	4976 (22.6)	4252 (12.8)		
Family stress	3027 (13.8)	2625 (7.9)		
Interpersonal stress	1723 (7.8)	1046 (3.1)		
Physical health problem	1349 (6.1)	7656 (23.0)		
Mental health problem	9336 (42.4)	15 613 (47.0)		
Other	708 (3.2)	740 (2.2)		

Abbreviation: AAU, acute alcohol use.

^a^ Poisoning from drugs, such as sleeping pills, painkillers, or other prescribed medications.

^b^ Poisoning from pesticides, insecticides, or herbicides.

^c^ Poisoning from gases such as carbon monoxide.

^d^ Self-inflicted injuries from sharp objects, fire, and/or moving objects.

^e^ Includes electrocution, removal of oxygen masks, and use of lethal poisons, such as pufferfish venom.

**Figure 1.**
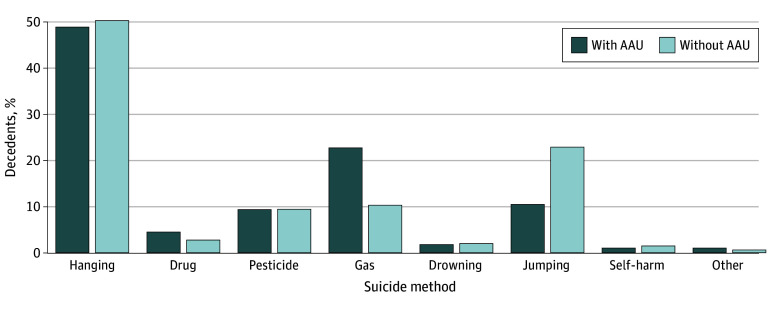
Methods of Suicide in Decedents With or Without AAU AAU indicates acute alcohol use; drug, poisoning from drugs such as sleeping pills, painkillers, or other prescribed medications; gas, poisoning from gases such as carbon monoxide; other, electrocution, removal of oxygen masks, or use of deadly poisons such as pufferfish venom; pesticide, poisoning from pesticides, insecticides, or herbicides; self-harm, self-inflicted injuries from sharp objects, fire, and/or moving objects.

The most common suicide method was hanging, accounting for 49.0% of individuals in the AAU group and 50.5% in the control group. In contrast, the second-most frequently used method differed between the groups. Gas poisoning was more prevalent in the AAU group (22.8%) compared with the control group (10.3%), while jumping was more common in the control group (22.9%) compared with the AAU group (10.5%) (Figure 1). In terms of presumed reasons for suicide, mental health problems were the most common in both groups (42.4% in the AAU group and 47.0% in the control group), followed by financial stress (22.6%) and family stress (13.8%) in the AAU group and physical health problems (23.0%) and financial stress (12.8%) in the control group.

### Factors Associated With AAU

Table 2 and the eTable in Supplement 1 summarize the hierarchical logistic regression models comparing suicide deaths with or without AAU. All models showed significant model fit, and the variables added at each step were associated with improvements in the models (step χ^2^_7_ = 8827.94 in model 2, step χ^2^_13_ = 2910.33 in model 3, and step χ^2^_49_ = 316.59 in model 4; all *P* < .001). In the final model, model 4, model χ^2^_82_ = 18 573.15 (*P* < .001) and a Nagelkerke *R*^2^ value of 0.39 indicate that the model explained 38.6% of the variance in the dependent variable.

**Table 2.** Hierarchical Logistic Regression Analysis of Factors Associated With Acute Alcohol Use

Independent variable	Model 1^a^			Model 2^b^			Model 3^c^			Model 4^d^		
	Unstandardized coefficient	OR (95% CI)	*P* value	Unstandardized coefficient	OR (95% CI)	*P* value	Unstandardized coefficient	OR (95% CI)	*P* value	Unstandardized coefficient	OR (95% CI)	*P* value
Sex^e^												
Male	0.59	1.81 (1.74-1.88)	<.001	0.38	1.46 (1.40-1.53)	<.001	0.30	1.36 (1.29-1.42)	<.001	0.32	1.37 (1.31-1.44)	<.001
Age, y^f^												
<20	−1.05	0.35 (0.30-0.41)	<.001	−1.07	0.34 (0.29-0.40)	<.001	−0.87	0.42 (0.36-0.49)	<.001	−1.18	0.31 (0.23-0.42)	<.001
30-39	0.25	1.28 (1.19-1.38)	<.001	0.16	1.17 (1.09-1.27)	<.001	0.12	1.12 (1.04-1.22)	<.01	0.18	1.20 (1.06-1.35)	<.01
40-49	0.14	1.15 (1.06-1.24)	<.001	−0.05	0.95 (0.88-1.03)	.23	−0.04	0.96 (0.89-1.05)	.39	0.01	1.01 (0.90-1.13)	.87
50-59	−0.05	0.95 (0.88-1.03)	.21	−0.40	0.67 (0.62-0.73)	<.001	−0.27	0.77 (0.70-0.84)	<.001	−0.31	0.73 (0.65-0.83)	<.001
60-69	−0.56	0.57 (0.52-0.62)	<.001	−0.91	0.40 (0.37-0.44)	<.001	−0.65	0.52 (0.47-0.58)	<.001	−0.74	0.48 (0.42-0.54)	<.001
70-79	−1.28	0.28 (0.25-0.31)	<.001	−1.49	0.23 (0.20-0.25)	<.001	−1.11	0.33 (0.29-0.37)	<.001	−1.35	0.26 (0.22-0.30)	<.001
≥80	−2.07	0.13 (0.11-0.14)	<.001	−2.13	0.12 (0.10-0.14)	<.001	−1.71	0.18 (0.16-0.21)	<.001	−2.13	0.12 (0.10-0.15)	<.001
Marital status^g^												
Separated	0.57	1.77 (1.62-1.94)	<.001	0.40	1.49 (1.35-1.65)	<.001	0.20	1.22 (1.10-1.35)	<.001	0.19	1.21 (1.09-1.35)	<.001
Not married	0.20	0.82 (0.77-0.86)	<.001	−0.13	0.88 (0.82-0.93)	<.001	−0.13	0.88 (0.83-0.94)	<.001	−0.13	0.88 (0.83-0.94)	<.001
Widowed	0.03	1.03 (0.94-1.13)	.50	0.01	1.01 (0.91-1.11)	.86	−0.17	0.84 (0.76-0.93)	<.001	−0.20	0.82 (0.74-0.91)	<.001
Divorced	0.48	1.62 (1.53-1.71)	<.001	0.39	1.47 (1.39-1.57)	<.001	0.27	1.31 (1.22-1.39)	<.001	0.26	1.30 (1.21-1.38)	<.001
Unknown	0.04	1.14 (0.96-1.13)	.36	0.06	1.06 (0.97-1.16)	.18	0	1.00 (0.91-1.09)	.96	0	1.00 (0.91-1.10)	.98
Psychiatric symptoms^h^												
Alcohol use disorder	NA	NA	NA	2.52	12.45 (11.64-13.32)	<.001	2.57	13.05 (12.17-13.98)	<.001	2.59	13.28 (12.38-14.24)	<.001
Psychosis	NA	NA	NA	−1.34	0.26 (0.24-0.29)	<.001	−1.02	0.36 (0.33-0.40)	<.001	−1.01	0.37 (0.33-0.40)	<.001
Manic symptoms	NA	NA	NA	−0.48	0.62 (0.54-0.70)	<.001	−0.37	0.69 (0.61-0.79)	<.001	−0.36	0.70 (0.62-0.80)	<.001
Depression	NA	NA	NA	−0.03	0.97 (0.93-1.02)	.19	0.12	1.13 (1.08-1.19)	<.001	0.13	1.14 (1.09-1.20)	<.001
Anxiety	NA	NA	NA	−0.11	0.90 (0.85-0.95)	<.001	−0.06	0.95 (0.89-1.01)	.07	−0.06	0.95 (0.89-1.01)	.08
Acute stress	NA	NA	NA	0.31	1.36 (1.27-1.46)	<.001	0.26	1.30 (1.21-1.40)	<.001	0.27	1.30 (1.21-1.40)	<.001
Other substance use	NA	NA	NA	1.04	2.82 (2.32-3.42)	<.001	1.08	2.94 (2.42-3.58)	<.001	1.08	2.96 (2.43-3.60)	<.001
Methods of suicide^i^												
Drug^j^	NA	NA	NA	NA	NA	NA	0.69	2.00 (1.79-2.23)	<.001	−0.02	0.98 (0.69-1.39)	.90
Pesticide^k^	NA	NA	NA	NA	NA	NA	0.63	1.87 (1.73-2.02)	<.001	−0.55	0.58 (0.36-0.93)	.02
Gas^l^	NA	NA	NA	NA	NA	NA	0.71	2.03 (1.92-2.15)	<.001	0.63	1.88 (1.61-2.20)	<.001
Drowning	NA	NA	NA	NA	NA	NA	0.21	1.23 (1.06-1.43)	<.01	0.16	1.17 (0.80-1.71)	.42
Jumping	NA	NA	NA	NA	NA	NA	−0.44	0.65 (0.61-0.69)	<.001	−0.29	0.75 (0.64-0.88)	<.001
Self-harm^m^	NA	NA	NA	NA	NA	NA	−0.23	0.80 (0.66-0.97)	.02	−0.72	0.49 (0.22-1.07)	.07
Other^n^	NA	NA	NA	NA	NA	NA	0.46	1.58 (1.25-1.99)	<.001	−0.06	0.94 (0.31-2.86)	.91
Presumed reason for suicide^o^												
Job stress	NA	NA	NA	NA	NA	NA	−0.25	0.78 (0.67-0.90)	<.01	−0.28	0.76 (0.65-0.88)	<.001
Financial stress	NA	NA	NA	NA	NA	NA	0.05	1.05 (0.93-1.19)	.45	0.03	1.03 (0.91-1.17)	.64
Family stress	NA	NA	NA	NA	NA	NA	0.38	1.47 (1.29-1.68)	<.001	0.36	1.44 (1.26-1.64)	<.001
Interpersonal stress	NA	NA	NA	NA	NA	NA	0.63	1.88 (1.63-2.18)	<.001	0.61	1.84 (1.59-2.13)	<.001
Physical health problems	NA	NA	NA	NA	NA	NA	−1.00	0.37 (0.32-0.42)	<.001	−1.01	0.36 (0.32-0.42)	<.001
Mental health problems	NA	NA	NA	NA	NA	NA	−0.32	0.73 (0.64-0.82)	<.001	−0.33	0.72 (0.64-0.81)	<.001
Age, y, and method of suicide^p^												
40-49 and Pesticide	NA	NA	NA	NA	NA	NA	NA	NA	NA	0.82	2.27 (1.33-3.88)	<.01
50-59 and Drug	NA	NA	NA	NA	NA	NA	NA	NA	NA	0.69	2.00 (1.31-3.05)	<.01
50-59 and Pesticide	NA	NA	NA	NA	NA	NA	NA	NA	NA	1.05	2.85 (1.72-4.71)	<.001
60-69 and Drug	NA	NA	NA	NA	NA	NA	NA	NA	NA	1.25	3.50 (2.21-5.56)	<.001
60-69 and Pesticide	NA	NA	NA	NA	NA	N A	NA	NA	NA	1.24	3.45 (2.09-5.69)	<.001
60-69 and Gas	NA	NA	NA	NA	NA	NA	NA	NA	NA	0.38	1.46 (1.15-1.85)	<.01
70-79 and Drug	NA	NA	NA	NA	NA	NA	NA	NA	NA	1.62	5.07 (3.21-8.01)	<.001
70-79 and Pesticide	NA	NA	NA	NA	NA	NA	NA	NA	NA	1.56	4.78 (2.90-7.86)	<.001
70-79 and Gas	NA	NA	NA	NA	NA	NA	NA	NA	NA	0.48	1.62 (1.20-2.19)	<.01
≥80 and Drug	NA	NA	NA	NA	NA	NA	NA	NA	NA	1.84	6.28 (3.53-11.17)	<.001
≥80 and Pesticide	NA	NA	NA	NA	NA	NA	NA	NA	NA	1.88	6.56 (3.86-11.13)	<.001
≥80 and Gas	NA	NA	NA	NA	NA	NA	NA	NA	NA	0.91	2.48 (1.52-4.04)	<.001

Abbreviations: NA, not applicable; OR, odds ratio.

^a^ 2-Log likelihood: 67 741.62; model χ^2^_13_ = 6518.29; step χ^2^ = NA; Nagelkerke *R*^2^ = 0.15.

^b^ 2-Log likelihood: 58 913.68; model χ^2^_20_ = 15 346.23; step χ^2^_7_ = 8827.94; Nagelkerke *R*^2^ = 0.33.

^c^ 2-Log likelihood: 56 003.35; model χ^2^_33_ = 18 256.56; step χ^2^_13_ = 2910.33; Nagelkerke *R*^2^ = 0.38.

^d^ 2-Log likelihood: 55 686.76; model χ^2^_82_ = 18 573.15; step χ^2^_49_ = 316.59; Nagelkerke *R*^2^ = 0.39.

^e^ Female was the reference category.

^f^ Aged 20 to 29 years was the reference category.

^g^ Married was the reference category.

^h^ No psychiatric symptoms was the reference category.

^i^ Hanging was the reference category.

^j^ Poisoning from drugs, such as sleeping pills, painkillers, or other prescribed medications.

^k^ Poisoning from pesticides, insecticides, or herbicides.

^l^ Poisoning from gases such as carbon monoxide.

^m^ Self-inflicted injuries from sharp objects, fire, and/or moving objects.

^n^ Includes electrocution, removal of oxygen masks, and use of lethal poisons, such as pufferfish venom.

^o^ Reasons were determined based on the guidelines provided by the Korea Foundation for Suicide Prevention. Investigators who reviewed police records identified reasons using the following criteria: (1) stressors that persisted in the decedent’s life before death, (2) the stressor that caused the most significant distress, and (3) the single factor most closely associated with the suicide. Other was the reference category.

^p^ Only significant results are presented. Full results are available in the eTable in Supplement 1. Aged 20 to 29 years and hanging was the reference category.

#### Demographic Variables

The association between demographic variables and AAU was examined using model 1. Male sex was associated with AAU in all 4 models (model 4: OR, 1.37 [95% CI, 1.31-1.44]). Individuals aged between 30 and 39 years showed higher odds of AAU than those in their 20s in all 4 models (model 4: OR, 1.20 [95% CI, 1.06-1.35]). In contrast, individuals younger than 20 years (model 4: OR, 0.31 [95% CI, 0.23-0.42]) and older than 50 years had lower odds of AAU. Among those aged 50 years or older, the odds of alcohol use decreased with age in model 4 (aged 50 to 59 years: OR, 0.73 [95% CI, 0.65-0.83]; aged 60 to 69 years: OR, 0.48 [95% CI, 0.42-0.54]; aged 70 to 79 years: OR, 0.26 [95% CI, 0.22-0.30]; and 80 years or older: OR, 0.12 [95% CI, 0.10-0.15]). Being separated (model 4: OR, 1.21 [95% CI, 1.09-1.35]) or divorced (model 4: OR, 1.30 [95% CI, 1.21-1.38]) was positively associated with AAU across all 4 models, while being unmarried (model 4: OR, 0.88 [95% CI, 0.83-0.94]) was associated with lower odds of alcohol use before suicide in all 4 models. Being widowed was associated with lower odds of AAU in models 3 (OR, 0.84 [95% CI, 0.76-0.93]) and 4 (OR, 0.82 [95% CI, 0.74-0.91]).

#### Psychiatric Symptoms

Psychiatric symptoms were included in model 2. The presence of AUD symptoms before death showed the highest odds of AAU among all variables in models 2, 3, and 4, with significantly higher odds of AAU (model 4: OR, 13.28 [95% CI, 12.38-14.24]). In model 4, other substance use (OR, 2.96 [95% CI, 2.43-3.60]), acute stress (OR, 1.30 [95% CI, 1.21-1.40]), and depressive symptoms (OR, 1.14 [95% CI, 1.09-1.20]) were also associated with AAU. However, the presence of psychosis (OR, 0.37 [95% CI, 0.33-0.40]) and manic symptoms (OR, 0.70 [95% CI, 0.62-0.80]) was associated with decreased odds of AAU.

#### Suicide Method and Reasons for Suicide

Suicide characteristics were included in model 3. Regarding suicide methods, gas poisoning showed the highest odds ratio among other methods (OR, 2.03 [95% CI, 1.92-2.15]). Drug poisoning (OR, 2.00 [95% CI, 1.79-2.23]), pesticide poisoning (OR, 1.87 [95% CI, 1.73-2.02]), drowning (OR, 1.23 [95% CI, 1.06-1.43]), and other methods (OR, 1.58 [95% CI, 1.25-1.99]) were also positively associated with AAU. Jumping (OR, 0.65 [95% CI, 0.61-0.69]) was associated with lower odds of AAU in model 3.

Building on these findings, model 4 examined the interaction between age and method; the results revealed that decedents who died by suicide at age 60 years or older were more likely to use drugs, pesticides, and gas poisoning as suicide methods. The ORs of these methods increased with age (aged 80 years or older: OR, 6.28 [95% CI, 3.53-11.17] for drug poisoning; OR, 6.56 [95% CI, 3.86-11.13] for pesticide poisoning; and OR, 2.48 [95% CI, 1.52-4.04] for gas poisoning).

**Figure 2.**
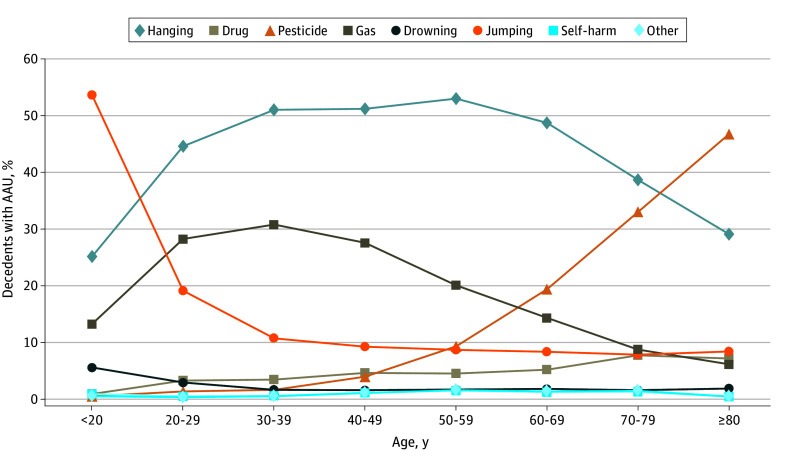
Methods of Suicide by Age in the Population With AAU AAU indicates acute alcohol use; drug, poisoning from drugs such as sleeping pills, painkillers, or other prescribed medications; gas, poisoning from gases such as carbon monoxide; other, electrocution, removal of oxygen masks, or use of deadly poisons such as pufferfish venom; pesticide, poisoning from pesticides, insecticides, or herbicides; self-harm, self-inflicted injuries from sharp objects, fire, and/or moving objects.

When the interactions between age and suicide method were controlled for, only gas poisoning showed a positive association with AAU (model 4: OR, 1.88 [95% CI, 1.61-2.20]), while the associations with drug poisoning, pesticide poisoning, and drowning became nonsignificant. Model 4 also showed jumping to be associated with lower odds of AAU (OR, 0.75 [95% CI, 0.64-0.88]). Additional χ^2^ tests revealed significant differences in suicide methods by age group within the AAU group. For example, 46.7% of people aged 80 years or older appeared to use pesticides, compared with less than 1% of people in their 20s (Figure 2).

Concerning the presumed reasons for suicide, in model 4, interpersonal stress (OR, 1.84 [95% CI, 1.59-2.13]) and family stress (OR, 1.44 [95% CI, 1.26-1.64]) were associated with AAU. Individuals whose presumed reason for suicide was job stress (OR, 0.76 [95% CI, 0.65-0.88]) or physical (OR, 0.36 [95% CI, 0.32-0.42) or mental (OR, 0.72 [95% CI, 0.64-0.81) health problems showed lower odds of AAU.

## Discussion

Using nationally representative data from South Korea, this cross-sectional study aimed to investigate the factors associated with AAU prior to suicide, with a specific focus on the characteristics of the suicide methods used. AAU is a significant risk factor for suicide, and understanding how it influences suicidal behavior is crucial for developing effective suicide interventions. The etiology of AAU-related suicide is multifaceted, involving demographic characteristics, psychiatric disorders such as AUD, and suicide-related factors.

In terms of demographics, hierarchical logistic regression results indicated that male and middle-aged individuals (aged 30 to 39 years) were more likely to have consumed alcohol before suicide. In contrast, female individuals, younger individuals (aged <20 years), and older adults (aged ≥50 years) had lower odds of AAU. This study further supports previous studies in which a high prevalence of AAU among male and middle-aged individuals is a widespread phenomenon observed in different cultural contexts. Previous studies from the US, Australia, New Zealand, and Kazakhstan have supported similar patterns, showing a higher prevalence of AAU in both middle-aged individuals and males.^6,8,14,35,36,37^

The presence of AUD was associated with AAU; individuals with AUD were significantly more likely (OR, 13.28 [95% CI, 12.38-14.24]) to exhibit AAU. Given that AUD is characterized by maladaptive patterns of alcohol consumption, typically involving frequent alcohol use, high odds of AAU were expected; notably, 86.5% of individuals with AUD showed AAU, suggesting a dual association of AUD and AAU with suicide, both in the long and short terms. Chronic alcohol use is not only associated with elevated suicide risk itself,^26,38^ it also exacerbates other suicide risk factors, such as depression,^39,40^ and negative life events, including marital dissolution,^41,42,43^ warranting critical need for early intervention and targeted prevention strategies. It is also important to note that only 33.8% of decedents with AAU had symptoms of AUD in this study, indicating that AAU frequently occurs in individuals without chronic alcohol use problems. These findings underscore the need for clinical attention to acute drinking behaviors in individuals at risk for suicide, whether with or without AUD.

An association between the method of suicide and AAU was also suggested, with drugs, pesticides, and gas poisoning showing significantly higher ORs (model 3), while jumping had a negative association. This contrasts with previous research that suggests a positive association between AAU and more lethal suicide methods, as methods such as drug, pesticide, and gas poisoning typically have lower case fatality rates.^17^ An interaction between age and method was also observed. Older decedents who consumed alcohol before they died by suicide had higher odds of using drugs, pesticides, and gas poisoning. The prevalence of pesticide poisoning increased with age, whereas that of jumping decreased. These findings align with those of previous studies indicating that older decedents who consumed alcohol before suicide were significantly more likely to die from drug use.^24^ Suicide among older adults associated with pesticide poisoning is likely due to the high proportion of older adults in agricultural sectors and their accessibility to pesticides in the environment.^44,45^ Drug and alcohol interactions, combined with preexisting physical illnesses, may result in more fatalities in older adults,^46^ contributing to the data showing a higher prevalence of drug, pesticide, and gas poisoning suicides in this age group. Taken together, it is possible that rather than AAU influencing the choice of the less lethal method, this less lethal method may interact with alcohol to increase the fatality of the outcome. These findings emphasize the need for tailored prevention efforts that address the multifaceted nature of AAU and its association with the lethality of suicide methods, particularly in older adults.

In contrast, hanging emerged as the most frequently used method of suicide across both AAU and control groups, consistent with prior research highlighting its lethality and widespread accessibility.^17,47^ The minimal variation in hanging between groups suggests that its use may be less influenced by acute alcohol consumption compared with other methods, such as gas poisoning or drug poisoning, which was associated with AAU. These findings underscore the need for prevention strategies that address the accessibility of hanging and its consistently high prevalence in suicide deaths worldwide.

Intriguingly, further analysis (model 4), which controlled for the interaction of age and method, showed that only gas poisoning (eg, charcoal-burning suicide) remained positively associated with AAU. Specifically, 22.8% of those in the AAU group used gas as a suicide method compared with only 10.3% of those without AAU. While AAU is widely conceptualized as a proximal risk factor that precedes suicide risk, an alternative framework complements this understanding by suggesting that alcohol may not only precede suicidal behavior but also may be deliberately consumed to facilitate it. Previous research has demonstrated that a significant subgroup of individuals intentionally consumes alcohol before a suicide attempt to numb their fear and pain.^48^ In this study, charcoal burning, which involves inhaling carbon monoxide in a confined space, accounted for most gas-poisoning suicides. This method requires considerable preparation, such as sealing off a space or entering a vehicle to produce lethal levels of carbon monoxide.^49^ Our findings may reflect individuals for whom alcohol is deliberately used to overcome psychologic barriers, such as fear or hesitation, enabling the execution of this planned and time-intensive method of suicide. Moreover, charcoal-burning suicides are culturally perceived as a painless method of dying,^50,51^ which may have contributed to an increase in overall suicide rates.^52,53,54^ Charcoal-burning suicides, especially when combined with alcohol, may have served as a practical factor that increases suicide capability, as described in the 3-step theory of suicide.^55^ Therefore, implementing policies such as tracking the simultaneous purchase of charcoal and alcohol or limiting the portrayal of this suicide method in the media could help reduce charcoal-burning suicides.

### Limitations

Despite its contributions, this study has several limitations. First, as the data were collected through psychological autopsies based on police reports, the accuracy and completeness of these reports may vary depending on the cooperation of bereaved family members, their knowledge of the deceased, and the subjective judgments of police officers and investigators, particularly for variables such as the presumed reasons for suicide. Second, the cross-sectional nature of the study limited its ability to infer a causal relationship between AAU and choice of suicide method. Longitudinal studies are required to understand the temporal relationship between AAU and suicide methods. Third, considering potential differences in patterns and contributing factors between nonfatal and fatal suicide attempts, future studies should include both suicide attempts and suicide deaths to provide a more comprehensive understanding of the relationship between AAU and suicide method selection. Finally, the findings are based on data from South Korea; additional research is necessary to determine the generalizability of these results to other cultural contexts.

## Conclusions

In this cross-sectional study of the association between AAU and suicide methods, gas poisoning, mainly through charcoal burning, was prominently associated with AAU, suggesting the possibility of deliberate alcohol consumption to facilitate suicide. Additionally, the interaction between age and suicide methods highlights that older adults who consumed alcohol before suicide were more likely to use less lethal methods, such as drugs, pesticides, or gas poisoning. These findings underscore the need for targeted policies aimed at monitoring and restricting alcohol access in high-risk situations for suicide. They also highlight the importance of providing clinical attention to drinking behaviors, even in individuals without chronic AUDs, as part of comprehensive suicide prevention efforts.

## Notes

Supplement 1. **eTable.** Full Results of Hierarchical Logistic Regression Analysis for Factors Associated With AAU

Supplement 2.
Data Sharing Statement
