# Supplementary material for: Acute Alcohol Use and Suicide
Source: JAMA Netw Open. 2025 Feb 24;8(2):e2461409. doi: 10.1001/jamanetworkopen.2024.61409 (PMC11851243; doi:10.1001/jamanetworkopen.2024.61409)
Supplement: Supplement 1. — eTable. Full Results of Hierarchical Logistic Regression Analysis for Factors Associated With AAU [file jamanetwopen-e2461409-s001.pdf]

Supplemental Online Content

Yim M, Kim H, Kim G, Hur JW. Acute alcohol use and suicide. *JAMA Netw Open*. 2025;8(2):e2461409. doi:10.1001/jamanetworkopen.2024.61409

eTable. Full Results of Hierarchical Logistic Regression Analysis for Factors Associated With AAU

This supplemental material has been provided by the authors to give readers additional information about their work.

© 2025 Yim M et al. *JAMA Network Open*.

eTable. Full Results of Hierarchical Logistic Regression Analysis for Factors Associated With AAU

							95% CI	
	B	*S.E.*	Wald	*df*	*P*	Exp (B)	Lower	Upper
**Model 1**								
Sex (ref. Female)								
Male	0.59	0.02	850.470	1	< .001	1.81	1.74	1.88
Age (ref. 20–29)								
< 20	-1.05	0.08	179.457	1	< .001	0.35	0.30	0.41
30–39	0.25	0.04	42.216	1	< .001	1.28	1.19	1.38
40–49	0.14	0.04	12.197	1	< .001	1.15	1.06	1.24
50–59	-0.05	0.04	1.571	1	.21	0.95	0.88	1.03
60–69	-0.56	0.04	160.649	1	< .001	0.57	0.52	0.62
70–79	-1.28	0.05	706.991	1	< .001	0.28	0.25	0.31
≥ 80	-2.07	0.06	1095.414	1	< .001	0.13	0.11	0.14
Marital Status (ref. Married)								
Separated	0.57	0.05	156.714	1	< .001	1.77	1.62	1.94
Not married	-0.20	0.03	51.260	1	< .001	0.82	0.77	0.86
Widowed	0.03	0.05	0.462	1	.50	1.03	0.94	1.13
Divorced	0.48	0.03	286.363	1	< .001	1.62	1.53	1.71
Unknown	0.04	0.04	0.846	1	.36	1.04	0.96	1.13
**Model 2**								
Sex (ref. Female)								
Male	0.38	0.02	284.061	1	< .001	1.46	1.40	1.53
Age (ref. 20–29)								
< 20	-1.07	0.08	175.889	1	< .001	0.34	0.29	0.40
30–39	0.16	0.04	15.914	1	< .001	1.17	1.09	1.27
40–49	-0.05	0.04	1.433	1	.23	0.95	0.88	1.03
50–59	-0.40	0.04	80.544	1	< .001	0.67	0.62	0.73
60–69	-0.91	0.05	347.112	1	< .001	0.40	0.37	0.44
70–79	-1.49	0.05	807.794	1	< .001	0.23	0.20	0.25
≥ 80	-2.13	0.07	1038.766	1	< .001	0.12	0.10	0.14
Marital Status (ref. Married)								
Separated	0.40	0.05	61.920	1	< .001	1.49	1.35	1.65
Not married	-0.13	0.03	18.223	1	< .001	0.88	0.82	0.93
Widowed	0.01	0.05	0.030	1	.86	1.01	0.91	1.11

© 2025 Yim M et al. *JAMA Network Open*.

eTable – continued

	B	*S.E.*	Wald	*df*	*P*	Exp(B)	95% CI Lower	Upper
Divorced	0.39	0.03	151.349	1	< .001	1.47	1.39	1.57
Unknown	0.06	0.05	1.818	1	.18	1.06	0.97	1.16
Psychiatric Symptoms (ref. No psychiatric symptoms)								
Alcohol use disorder	2.52	0.03	5360.844	1	< .001	12.45	11.64	13.32
Psychosis	-1.34	0.05	753.408	1	< .001	0.26	0.24	0.29
Manic Symptoms	-0.48	0.07	53.202	1	< .001	0.62	0.54	0.70
Depression	-0.03	0.02	1.712	1	.19	0.97	0.93	1.02
Anxiety	-0.11	0.03	12.519	1	< .001	0.90	0.85	0.95
Acute stress	0.31	0.04	75.621	1	< .001	1.36	1.27	1.46
Other substance abuse	1.04	0.10	110.503	1	< .001	2.82	2.32	3.42
**Model 3**								
Gender (ref. Female)								
Male	0.30	0.02	161.932	1	< .001	1.36	1.29	1.42
Age (ref. 20–29)								
< 20	-0.87	0.08	109.298	1	< .001	0.42	0.36	0.49
30–39	0.12	0.04	7.820	1	.005	1.12	1.04	1.22
40–49	-0.04	0.04	0.731	1	.39	0.96	0.89	1.05
50–59	-0.27	0.05	32.838	1	< .001	0.77	0.70	0.84
60–69	-0.65	0.05	160.169	1	< .001	0.52	0.47	0.58
70–79	-1.11	0.06	391.395	1	< .001	0.33	0.29	0.37
≥ 80	-1.71	0.07	585.492	1	< .001	0.18	0.16	0.21
Marital Status (ref. Married)								
Separated	0.20	0.05	14.424	1	< .001	1.22	1.10	1.35
Not married	-0.13	0.03	15.789	1	< .001	0.88	0.83	0.94
Widowed	-0.17	0.05	10.840	1	< .001	0.84	0.76	0.93
Divorced	0.27	0.03	65.160	1	< .001	1.31	1.22	1.39
Unknown	0.00	0.05	0.003	1	.96	1.00	0.91	1.09
Psychiatric Symptoms (ref. none)								
Alcohol use disorder	2.57	0.04	5260.194	1	< .001	13.05	12.17	13.98
Psychosis	-1.02	0.05	395.441	1	< .001	0.36	0.33	0.40
Manic Symptoms	-0.37	0.07	29.493	1	< .001	0.69	0.61	0.79

© 2025 Yim M et al. *JAMA Network Open*.

eTable – continued

	B	*S.E.*	Wald	*df*	*P*	Exp(B)	95% CI	
							Lower	Upper
Depression	0.12	0.02	24.999	1	< .001	1.13	1.08	1.19
Anxiety	−0.06	0.03	3.086	1	.07	0.95	0.89	1.01
Acute stress	0.26	0.04	49.913	1	< .001	1.30	1.21	1.40
Other substance abuse	1.08	0.10	116.430	1	< .001	2.94	2.42	3.58
Methods of suicide (ref. Hanging)								
Drug[a]	0.69	0.06	148.805	1	< .001	2.00	1.79	2.23
Pesticide[b]	0.63	0.04	247.367	1	< .001	1.87	1.73	2.02
Gas[c]	0.71	0.03	601.519	1	< .001	2.03	1.92	2.15
Drowning	0.21	0.08	7.717	1	.005	1.23	1.06	1.43
Jumping	−0.44	0.03	184.852	1	< .001	0.65	0.61	0.69
Self-harm[d]	−0.23	0.10	5.117	1	.024	0.80	0.66	0.97
Other[e]	0.46	0.12	14.639	1	< .001	1.58	1.25	1.99
Presumed reason for suicide[f] (ref. No psychiatric symptoms)								
Job stress	−0.25	0.08	10.983	1	.001	0.78	0.67	0.90
Financial stress	0.05	0.06	0.572	1	.45	1.05	0.93	1.19
Family stress	0.38	0.07	32.534	1	< .001	1.47	1.29	1.68
Interpersonal stress	0.63	0.07	73.485	1	< .001	1.88	1.63	2.18
Physical health problems	−1.00	0.07	211.126	1	< .001	0.37	0.32	0.42
Mental health problems	−0.32	0.06	26.133	1	< .001	0.73	0.64	0.82
**Model 4**								
Gender (ref. Female)								
Male	0.32	0.02	173.018	1	< .001	1.37	1.31	1.44
Age (ref. 20–29)								
< 20	−1.18	0.16	56.623	1	< .001	0.31	0.23	0.42
30–39	0.18	0.06	9.513	1	.002	1.20	1.06	1.35
40–49	0.01	0.06	0.025	1	.87	1.01	0.90	1.13
50–59	−0.31	0.06	26.194	1	< .001	0.73	0.65	0.83
60–69	−0.74	0.07	121.370	1	< .001	0.47	0.42	0.54
70–79	−1.35	0.08	316.797	1	< .001	0.26	0.22	0.30
≥ 80	−2.13	0.11	387.142	1	< .001	0.12	0.10	0.15

© 2025 Yim M et al. *JAMA Network Open*.

eTable – continued

	B	*S.E.*	Wald	*df*	*P*	Exp(B)	95%CI Lower	Upper
Marital Status (ref. Married)								
Separated	0.19	0.05	13.411	1	< .001	1.21	1.09	1.35
Not married	−0.13	0.03	14.837	1	< .001	0.88	0.83	0.94
Widowed	−0.20	0.05	14.086	1	< .001	0.82	0.74	0.91
Divorced	0.26	0.03	60.996	1	< .001	1.30	1.21	1.38
Unknown	0.00	0.05	0.000	1	.98	1.00	0.91	1.10
Psychiatric Symptoms (ref. No psychiatric symptoms)								
Alcohol use disorder	2.59	0.04	5233.297	1	< .001	13.28	12.38	14.24
Psychosis	−1.01	0.05	384.804	1	< .001	0.37	0.33	0.40
Manic symptoms	−0.36	0.07	27.754	1	< .001	0.70	0.62	0.80
Depression	0.13	0.02	27.834	1	< .001	1.14	1.09	1.20
Anxiety	−0.06	0.03	3.179	1	.08	0.95	0.89	1.01
Acute stress	0.27	0.04	51.150	1	< .001	1.30	1.21	1.40
Other substance abuse	1.08	0.10	117.164	1	< .001	2.96	2.43	3.60
Methods of suicide (ref. Hanging)								
Drug[a]	−0.02	0.18	0.015	1	.90	0.98	0.69	1.39
Pesticide[b]	−0.55	0.24	5.195	1	.02	0.58	0.36	0.93
Gas[c]	0.63	0.08	63.728	1	< .001	1.88	1.61	2.20
Drowning	0.16	0.19	0.646	1	.42	1.17	0.80	1.71
Jumping	−0.29	0.08	12.681	1	< .001	0.75	0.64	0.88
Self-harm[d]	−0.72	0.40	3.248	1	.07	0.49	0.22	1.07
Other[e]	−0.06	0.57	0.013	1	.91	0.94	0.31	2.86
Presumed reason for suicide[f] (ref. Other)								
Job stress	−0.28	0.08	13.116	1	< .001	0.76	0.65	0.88
Financial stress	0.03	0.06	0.223	1	.64	1.03	0.91	1.17
Family stress	0.36	0.07	29.029	1	< .001	1.44	1.26	1.64
Interpersonal stress	0.61	0.07	68.313	1	< .001	1.84	1.59	2.13
Physical health problems	−1.01	0.07	214.236	1	< .001	0.36	0.32	0.42
Mental health problems	−0.33	0.06	27.383	1	< .001	0.72	0.64	0.81

© 2025 Yim M et al. *JAMA Network Open*.

eTable – continued

	B	*S.E.*	Wald	*df*	*P*	Exp(B)	95%CI Lower	95%CI Upper
Age × Method of suicide (ref. 20s, Hanging)								
< 20 × Drug	−0.15	0.79	0.035	1	.85	0.86	0.18	4.08
< 20 × Pesticide	−0.39	1.08	0.132	1	.72	0.68	0.08	5.60
< 20 × Gas	0.44	0.28	2.426	1	.12	1.55	0.89	2.71
< 20 × Drowning	0.47	0.42	1.238	1	.27	1.59	0.70	3.63
< 20 × Jumping	0.28	0.20	1.990	1	.16	1.32	0.90	1.95
< 20 × Self-harm	24.15	23816.614	0.000	1	1.00	3.081×10^{10}	0.00	
< 20 × Other	−0.21	1.83	0.013	1	.91	0.81	0.02	29.53
30–39 × Drug	0.14	0.23	0.348	1	.56	1.15	0.73	1.80
30–39 × Pesticide	0.16	0.30	0.289	1	.59	1.18	0.65	2.13
30–39 × Gas	−0.14	0.10	1.956	1	.16	0.87	0.72	1.06
30–39 × Drowning	−0.01	0.28	0.002	1	.96	0.99	0.57	1.71
30–39 × Jumping	−0.17	0.11	2.398	1	.12	0.84	0.68	1.05
30–39 × Self-harm	−0.12	0.51	0.059	1	.81	0.88	0.33	2.39
30–39 × Other	0.13	0.68	0.034	1	.85	1.13	0.30	4.31
40–49 × Drug	0.42	0.22	3.811	1	.05	1.52	1.00	2.32
40–49 × Pesticide	0.82	0.27	9.052	1	< .001	2.27	1.33	3.87
40–49 × Gas	−0.10	0.10	1.005	1	.32	0.91	0.75	1.10
40–49 × Drowning	−0.15	0.26	0.337	1	.56	0.86	0.52	1.42
40–49 × Jumping	−0.24	0.11	5.050	1	.02	0.79	0.64	0.97
40–49 × Self-harm	0.13	0.45	0.084	1	.77	1.14	0.47	2.77
40–49 × Other	0.36	0.62	0.336	1	.56	1.43	0.42	4.86
50–59 × Drug	0.69	0.22	10.388	1	< .001	2.00	1.31	3.05
50–59 × Pesticide	1.05	0.26	16.643	1	< .001	2.85	1.72	4.71
50–59 × Gas	0.22	0.10	4.541	1	.03	1.24	1.02	1.52
50–59 × Drowning	0.18	0.25	0.499	1	.48	1.20	0.73	1.97
50–59 × Jumping	−0.22	0.11	4.109	1	.04	0.80	0.65	0.99
50–59 × Self-harm	0.77	0.44	3.063	1	.08	2.15	0.91	5.09
50–59 × Other	0.34	0.60	0.311	1	.58	1.40	0.43	4.59
60–69 × Drug	1.25	0.24	28.257	1	< .001	3.50	2.21	5.56
60–69 × Pesticide	1.24	0.26	23.553	1	< .001	3.45	2.09	5.69
60–69 × Gas	0.38	0.12	9.571	1	< .001	1.46	1.15	1.85

© 2025 Yim M et al. *JAMA Network Open*.

eTable – continued

							95%CI	
	B	*S.E.*	Wald	*df*	*P*	Exp(B)	Lower	Upper
60–69 × Drowning	0.05	0.29	0.032	1	.86	1.05	0.60	1.85
60–69 × Jumping	-0.30	0.12	5.606	1	.02	0.74	0.58	0.95
60–69 × Self-harm	0.72	0.47	2.353	1	.13	2.05	0.82	5.11
60–69 × Other	0.82	0.64	1.610	1	.20	2.26	0.64	7.98
70–79 × Drug	1.62	0.23	48.582	1	< .001	5.07	3.21	8.01
70–79 × Pesticide	1.56	0.25	37.875	1	< .001	4.78	2.90	7.86
70–79 × Gas	0.48	0.15	9.854	1	.002	1.62	1.20	2.19
70–79 × Drowning	0.27	0.32	0.700	1	.43	1.31	0.70	2.45
70–79 × Jumping	-0.20	0.14	1.884	1	.17	0.82	0.62	1.09
70–79 × Self-harm	1.09	0.49	4.914	1	.03	2.96	1.13	7.75
70–79 × Other	1.68	0.66	6.440	1	.012	5.37	1.47	19.69
≥ 80 × Drug	1.84	0.29	38.971	1	< .001	6.28	3.53	11.17
≥ 80 × Pesticide	1.88	0.27	48.574	1	< .001	6.56	3.86	11.13
≥ 80 × Gas	0.91	0.25	13.311	1	< .001	2.48	1.52	4.04
≥ 80 × Drowning	0.35	0.42	0.666	1	.41	1.41	0.62	3.25
≥ 80 × Jumping	0.02	0.20	0.014	1	.91	1.02	0.69	1.53
≥ 80 × Self-harm	-0.13	0.88	0.022	1	.88	0.88	0.16	4.94
≥ 80 × Other	0.53	0.95	0.312	1	.58	1.70	0.26	11.00

Note. AAU = Acute alcohol use; Drug[a] = Poisoning from drugs such as sleeping pills, painkillers, and other prescribed medications; Pesticide[b] = Poisoning from pesticides, insecticides, and herbicides; Gas[c] = Poisoning from gases such as carbon dioxide; Self-harm[d] = Self-inflicted injuries from sharp objects, fire, and/or moving objects; Other[e] = Examples of other methods, including death by electrocution, removal of oxygen masks, and use of lethal poisons, such as pufferfish venom.

Presumed reason for suicide[f]: The presumed reasons for suicide were determined based on the guidelines provided by the Korean Foundation for Suicide Prevention. Investigators who reviewed police records identified these reasons using the following criteria: (1) stressors that persisted in the decedent's life before death, (2) the stressor that caused the most significant distress, and (3) the single factor most closely associated with the suicide.

© 2025 Yim M et al. *JAMA Network Open*.
