# Supplementary material for: Acute Alcohol Use and Suicide
Source: JAMA Netw Open. 2025 Feb 24;8(2):e2461409. doi: 10.1001/jamanetworkopen.2024.61409 (PMC11851243; doi:10.1001/jamanetworkopen.2024.61409)
Supplement: Supplement 2. — Data Sharing Statement [file jamanetwopen-e2461409-s002.pdf]

Data Sharing Statement

Yim. Acute Alcohol Use and Suicide. *JAMA Netw Open*. Published February 24, 2025.
doi:10.1001/jamanetworkopen.2024.61409

Data
Data available: No

Additional Information
Explanation for why data not available: This data is accessible through in-person visits by individuals authorized by the Korea Foundation for Suicide Prevention. Website: https://www.kfsp.or.kr/
